# Caries of the Teeth

**Published:** 1856-01

**Authors:** J. Taft


### ARTICLE X.

### *Caries of the Teeth.* By J. Taft, D. D. S.

The teeth occupy a prominent position in the animal organization; in regard to the human economy, this is especially true. The well-being of the entire organization depends much upon the perfect performance of the functions of the teeth.

The teeth of the inferior animals seem to have their whole design fulfilled in the acts of mastication and offensive and defensive operations. But in man the offices of the teeth are more numerous.

Mastication may be regarded as the primary and most important. This is the first step in the process of nutrition, and a failure here is not remedied elsewhere, it is here that the imperfect work of the teeth is transferred to other organs, to be by them accomplished, so far as they can, in addition to their legitimate work. Thus, that which should be thoroughly executed, is imperfectly done; other organs are over-taxed; and thus rendered inefficient for the proper performance of their appropriate work. These organs then, suffer from over work, and they with all the other organs from want of proper nourishment.

Man alone is endowed with the power of speech. The manifestation of this faculty is through a set of organs beautifully adapted to the purpose. All important among these we find a perfect well arranged set of teeth. He only who has a perfect set of teeth can control and modulate his voice, so as to exhibit language in its native beauty.

The teeth aid in giving expression and beauty to the countenance. They impart beauty and symmetry to the immediate vicinity, giving a fullness and boldness of outline, that never exists after their removal. In color they contrast admirably with the part about them. In the manifestation of the mind through the human countenance the teeth play an important part.

Notwithstanding the teeth are so important in the human economy, and though their functions are so various, and of such magnitude, they are neglected—they are subject to positive violence. Very few give the requisite attention to the teeth to preserve them from the injurious agents.

Owing to the artificial manner of living, the frequent impairment of health, this is often very difficult to do.

The teeth are subjected to positive violence in a variety of ways; crushing or biting hard substances, sustaining weights, violent percussion, sudden extremes of temperature, bungling dental operations, etc.

The dentine is effected by caries more frequently, than by any other form of disease. It is fatal in its tendency and of very frequent occurrence, scarcely any who have attained the age of maturity are free from its ravages.

It is a disease against which the resisting powers can make but a feeble stand; and one in which the recuperative powers can avail nothing. The opinion is maintained by some that softened dentine does in many cases regain its natural density. This cannot be, unless the softened part retains its vitality. Any agent possessing power sufficient to decompose the dentine will destroy its vitality. What is that decomposition? It is either a want of ability on the part of the vital power to maintain the integrity of the organic structure, or the action of some agent having an affinity for some part of the dentine, more potent than the maintaining vital force. In either case the vitality is destroyed. In an organized structure the removal of one of its component parts occasions the loss of vitality of the remaining part.

Caries makes its attack first upon the dentine and progresses most rapidly in the direction of the tubuli. There are variations from this, an example of which we have in the large superficial caries upon the labial surfaces of the superior incisors. It also in many cases progresses rapidly immediately beneath the enamel.

Portions of the dentine imperfectly protected by the enamel, either on account of its injured condition, or from imperfect formation, are liable to be attacked by caries. Also points that by their location, or any unfavorable circumstance retain injurious agents in contact with the tooth, are very liable to decay.

The attack and progress of caries is modified by the constitution of the teeth.

They may be defective either originally or accidentally. Originally defective would extend to all the teeth of the same person. Accidental defection might exist only in some of the teeth in the same mouth, and these only at particular points. These conditions are peculiarly favorable for the attack of caries. When the whole crown of the tooth is imperfectly organized, decay will progress with uniform rapidity, under the influence of the agent producing it, till the entire crown is destroyed. When there are only portions of the tooth imperfectly organized they will be attacked by caries; after a time its progress becomes retarded, and in some cases checked altogether.

There are other circumstances that modify the progress of caries. A change of the condition or character of the agents producing it; the increased or diminished amount of such agents.

The progress of caries will also be goverend somewhat by the age of the person upon whose teeth it makes the attack. The teeth exhibit almost an infinite variety in regard to constitution. The relative proportion of their constituents is exceedingly various, even in persons of the same age. There is a continual change going on in the relative amount of the constituents of dentine as the person advances in age. The calcareous elements increasing and the annual decreasing. A proper relative amount of elements may be elaborated and yet a defective organization exist. This arises from an inability of the organizing power; or a want of arrangement and combination of the materials. The state of things is dependent entirely upon accidental causes. In vital energy, the teeth exhibit great diversity. This corresponds with, and is dependent upon, to some extent, the vital energy of the general constitution.

Dead dentine is decomposed more readily than the living, hence the conclusion that vitality resists caries; and the resistance corresponds with the vigor of that vitality.

The points most frequently attacked by caries, are the approximal surfaces of the teeth, the indentations and fissures upon the masticating surfaces of the molars and bicuspids, along, or at the termination of the longitudinal depressions upon the buccal and palatal walls of the molars, and at the necks of the teeth at the termination of the enamel. Upon the approximal surfaces of the teeth, the enamel is thinner than elsewhere; and the situation is one peculiarly favorable for the concentration and retention of injurious agents. The union of the enamel in the fissures and indentures of the crowns of the molars is frequently imperfect; and thus there is a way of entrance for vitiated fluids to the dentine. Decay is sooner exhibited at the termination or intersection of these fissures, than at any intermediate point.

The indentations or grooves upon the sides of the teeth are usually attacked by caries at that point next to the neck of the teeth. Caries, less frequent than in the above cases is exhibited at the necks of the teeth just beneath the border of the enamel; here it burrows under the enamel, though its transverse extension is greatest. The order in which the elements are removed is governed by the nature of the agent producing the decomposition. Usually the decomposition is produced by some agent having an affinity for the calcareous elements sufficiently strong to destroy the texture of the dentine and remove the earthy portion. The acids having an affinity for the lime of the dentine produces its decomposition in this manner. When the decay is thus produced, the portion remaining in the cavity is soft, and approximates the gelatinous condition, correspondent to the amount of calcareous material removed. In this kind of decay we find it soft and tenacious. Agents of a different character from those referred to, often produce decay. Alkalies will act upon the animal portion of the dentine, and remove it. In caries thus produced the residue is friable and chalk-like. In other cases the constituents are removed simultaneously. Nitric acid will produce the entire breaking up of both the earthy and animal constituents.

Death of the dentine generally produces decay. It is more easily decomposed after the vitality is destroyed. There are apparent exceptions to this. The dentine beyond the decay may be in an inflamed and irritable condition, so that contact with the decayed part will produce pain; in this way we may be lead to conclude that the softened dentine is sensitive, when such is not the case. It is maintained that there are cases in which the partially decomposed dentine is sensitive, supposing that a small portion of the calcareous elements may be removed, and yet the filiments of the nerve ramifying the part not to be destroyed.

The progress of caries is far more rapid in the crowns of the teeth than in the roots. The crowns are more exposed to the influences of external injuries. It is true that the crowns are covered by enamel designed to shield the dentine from injury, but it is often defective, and agents are concentrated upon it that it cannot resist, even when it is perfect, so that the enamel itself is sometimes decomposed. The roots possess a higher degree of vitality than the crowns, and the resistance to the encroachments of decay is correspondingly greater, hence we often find roots free from decay and solid, the crowns of which have been removed by rapid decay.

Injurious substances are sometimes pressed into contact with the dentine through defective points of the enamel, or under its projections, and thus retained till its mischievous action is accomplished.

It is maintained by some writers that caries is contagious. Dr. Koecker was of this opinion. The question arises here, is there any property in the decayed dentine capable of producing the same condition in the healthy dentine of another tooth?

The residue of decayed dentine is, in the soft decay, the animal elements, with a slight portion of earthy material; and in decay, in which the gelatinous portion is removed, the remainder is chalk-like, consisting mainly, of phosphate of lime. In neither of these is there anything that can possibly operate on the healthy dentine. There is one thing here worthy of consideration, and which has probably led to the mistaken notion that caries is contagious. The decayed dentine will absorb and retain fluids that will act on the sound dentine. And when the decay is on the approximal portion of the tooth, two teeth are subject to the exciting cause, so that, so far as the chemical action of the agent is concerned, the two teeth are alike subject to its operation.

It is but seldom that two teeth thus situated are both in the same stage of decay. This arises, principally, from the difference in their constitution.

The decay of the teeth, in pairs, has also been attributed to the contagious character of the disease. This, however, results from the fact, that the pairs of teeth are formed at the same time; and are subject to the same influences in their formation, and hence are constituted alike; and if one is defective the other will be in a similar condition. When there is a vitiation of the saliva, or mucus, they will be similarly affected. In no common acceptation of the term contagious, can it be applied to caries of the teeth. The color of caries is exceedingly various. It is found in color the same as the healthy dentine, and presenting every intermediate shade, from that to a jet black. The rapidity of decay may be determined by its color; it progresses less rapidly as the color becomes darker, so that, when the decay is almost stationary, the affected portion is black. The shades of color are differently marked by different writers. Koecker enumerates five, others seven, and so on. Three are sufficient for our purpose—*white*, *brown* and *black*. The sensitiveness of the dentine is greatest in teeth affected with the light colored decay. The sensitiveness usually decreases as the color increases. There are exceptions to this. We occasionally find teeth, the decay of which is dark, and yet quite sensitive. The light colored decay is more difficult to arrest than the dark. Dark, or black, decay is easily arrested, while the white is arrested with much difficulty. Filling, in many cases of this kind, seems scarcely to retard the progress of the decay; while in the brown, or black, decay, by proper filling, the progress of the decay is checked altogether. The cause of the dark color of caries is not perfectly comprehended. It is doubtless a deposit upon the decayed part, and it is most probably a metallic oxyd. In the saliva and mucus are found *iron*, *sodium*, *potassium*, and *calcium*, in several combinations.

The opinion is entertained by some, that this deposit protects the dentine from the influence of injurious agents. This is most probably not the case, at least to any perceivable extent. If this does serve as a protection, the removal of the discolored portion would subject the dentine to a renewed attack of caries, which experience assures us it does not do, but after some considerable time it assumes the dark hue again.

Those who maintain that the dentine is protected by the deposit, refer to the fact, that a deposit of oxyd of silver may be made upon decay of a light color, by the use of nit. arg., and that after such deposit is made, the progress of the decay is retarded. This, however, is more probably occasioned by a change in the character of the decay, and not because it is protected by the coating of oxyd of silver.

There is commonly some sensibility resulting from caries of the teeth. It does not often amount to pain, but is rather a sense of uneasiness; yet when anything is brought into contact with the sensitive dentine, as sudden changes of temperature, acids, &c., intense pain may be produced.

Dr. Koecker remarks, that caries is more tender in its first stages. Dr. Cone remarks, that when a tooth is attacked by caries, sensibility is increased. The surface of the dentine, or that part united to the enamel, is susceptible of the highest sensibility. At the point of union of the dentine with the enamel, is the termination of the nerve fibrils that pervade the dentine. These terminations possess a greater sensibility, both in a healthy and diseased state, than along the body of the fibrils. When there is inflammation of dentine, intense pain may be produced by the contact of an instrument, in a cavity of decay, at the line of union of the dentine with the enamel, and very little sensibility manifested elsewhere in the cavity. Sensibility of a uniform character sometimes pervades all parts of the cavity; at other times it may be very intense at one point, and very slight or not exist at all at any other point in the cavity. A thin lamina of the dentine lining the entire cavity, may be uniformly sensitive, and in some cases the sensibility will involve the entire body of the dentine.

By means of the sensibility, warning is transmitted to the pulp, and it throws out osseous material with increased energy, and thus a process of filling up the natural cavity of the tooth is established, that the decay may not encroach upon the nerve. This warning may be transmitted to the pulp to some extent, though there be no increase of sensibility.

The sensibility is modified by the character of the teeth, and the nature of the decay, and the state of the constitution of the patient. The teeth of the same person will be more sensitive at one time than at another, owing to the irritability of the nervous system. Those teeth that decay most rapidly, are usually most sensitive. There are some teeth in which the vitality is destroyed considerably in advance of the decay; and in such cases there is no sensibility at all. Except in such cases as last mentioned, the most rapid and whitest decay is most sensitive. There is much less sensibility in the brown, and scarcely any in the black decay.

Sensibility exists in all degrees, from the mildest to the most intense. It is very various in the teeth of the same person, at the same period; owing to their different susceptibility, and the difference in exposure to irritating agents. It is more common and more severe in young persons.—*Dental Register.*

